# Supplementary material for: ProteinGLUE multi-task benchmark suite for self-supervised protein modeling
Source: Sci Rep. 2022 Sep 26;12:16047. doi: 10.1038/s41598-022-19608-4 (PMC9512797; doi:10.1038/s41598-022-19608-4)
Supplement: Supplementary file 1 — Supplementary Information. [file 41598_2022_19608_MOESM1_ESM.pdf]

Supplementary information for ProteinGLUE: A multi-task benchmark suite for self-supervised protein modeling.

Henriette Capel[1,+], Robin Weiler[1,+], Maurits Dijkstra[1,+], Reinier Vleugels[1], Peter Bloem[1], K. Anton Feenstra[1,*]

[1] Vrije Universiteit, Informatics Institute, Amsterdam, 1081 HV, Netherlands
[+] these authors contributed equally to this work

Technical implementation details

Tensorflow format of datasets

The protein sequences and their corresponding labels were extracted from all the fine-tuning data sets and converted to a `TFRecord` file using `TFRecordWriter` (`www.tensorflow.org/api_docs/python/tf/io/TFRecordWriter`). The `TFrecord` format stores sequences and binary records. During the transformation the `tf.train.Example` message is used for the `""string": value mapping"`. This mapping can except the feature types bytes, float and int64. The protein sequence is therefore converted to a bytes list. The absolute solvent accessibility values is converted to a float. All other prediction tasks are stored as integers. The fine-tuning tasks SS3, SS8, BUR, PPI and EPI are binary values. For the hydrophobic patch prediction the patches were ranked per protein based on the size of the patch. Therefore, the patch value was indicated as integer.

Negative results

We tried using a reduced target alphabet of only 12 tokens for some of our experiments within the pre-training procedure because there is a lot of variation in the sequences possible for a protein with a given shape.[1,2] Hence, we tested whether mapping these similar amino acids to the same tokens would improve model performance since we did not want to penalize the model for predicting an amino acid which is largely the same in a protein structural context as the strictly correct one in that specific sequence. However, what we found was that even though using a reduced target alphabet obviously increased the performance metrics on the pre-training task, we did not observe a notable difference in performance on the downstream tasks.

References

1. Illergård, K., Ardell, D. H. & Elofsson, A. Structure is three to ten times more conserved than sequence—a study of structural response in protein cores. *Proteins: Struct. Funct. Bioinforma.* **77**, 499–508 (2009).

2. Murphy, L. R., Wallqvist, A. & Levy, R. M. Simplified amino acid alphabets for protein fold recognition and implications for folding. *Protein engineering* **13**, 149–152 (2000).

Figures & Tables

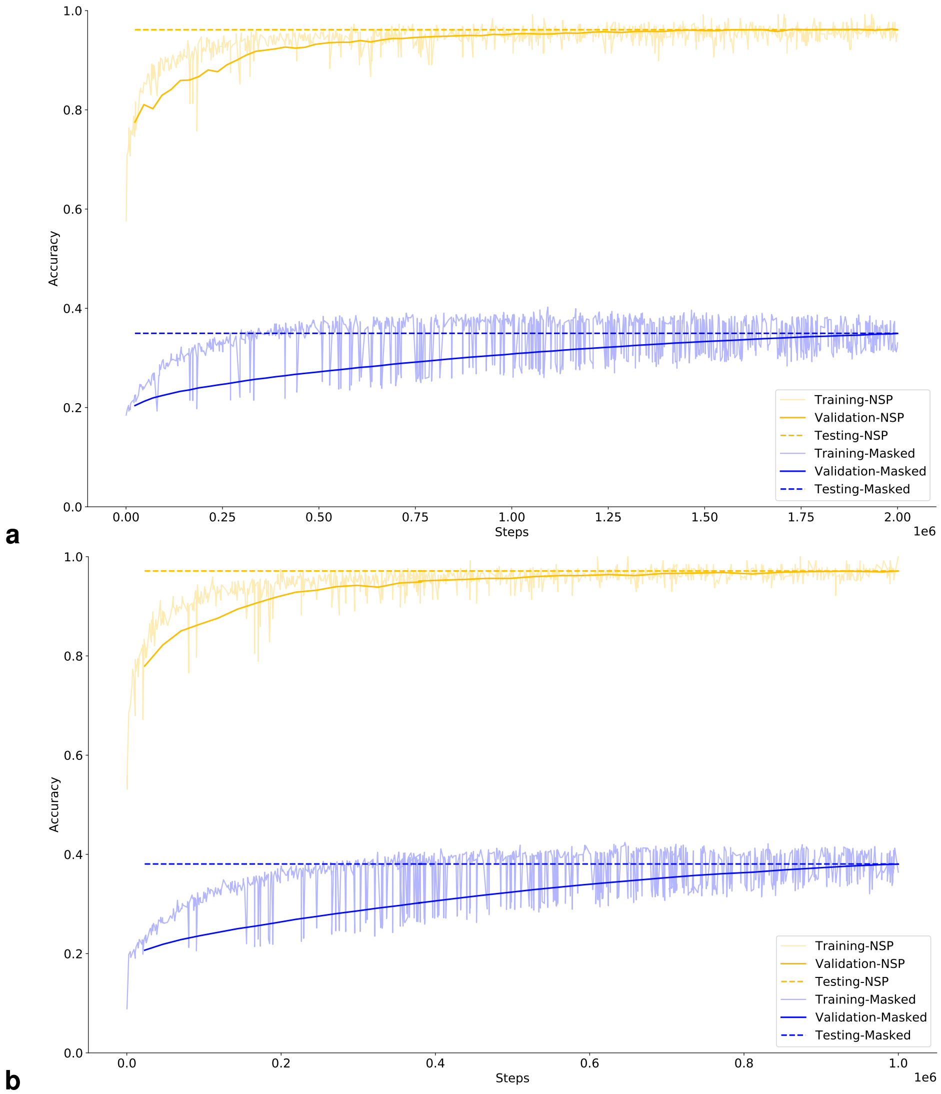

Figure 1. *Accuracy versus training steps* for masked token (blue) and next sentence (yellow) prediction on training (per batch) and validation (per epoch) data of (**a**) BERT `medium` and (**b**) BERT `base` pre-training runs. Dashed lines indicate the final testing data accuracy.

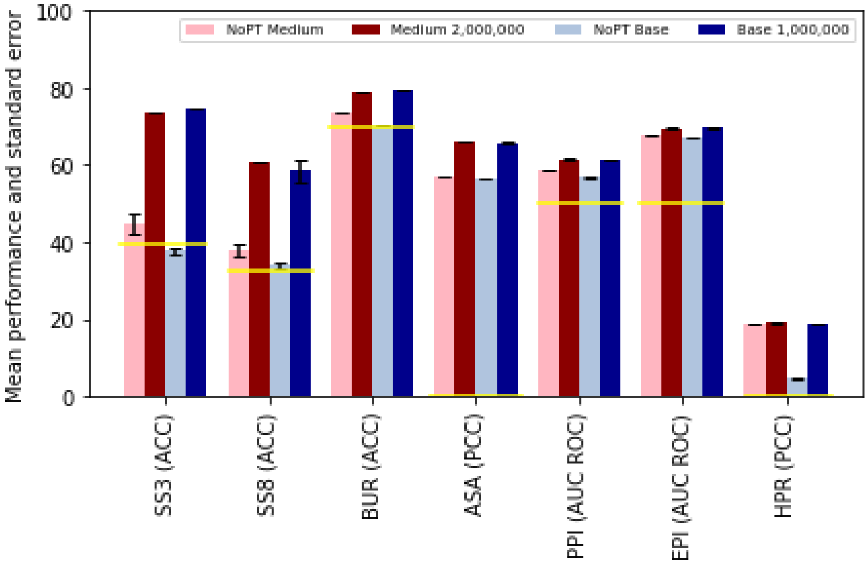

Figure 2. *Improved performance on fine-tuning tasks with pre-trained models.* Prediction performances of the benchmark validation set, for a `medium` model without the pre-training step (pink), `medium` model including a pre-trained model until step 2 000 000 (dark red), `base` model without the pre-training step (grey), and `base` model including a pre-trained model until step 1 000 000 (dark blue). The yellow lines indicate the performance of a random or majority-class baseline. All models are trained ten times on their selected set of hyperparameters after which the mean performance and standard error is determined.

Table 1. *The results of tuning the hyperparameters in pretraining for the converged* `medium` *and* `base` *models.*

Model	Pre-training task	Batch size	Learning rate	Dropout rate	mean performance
`medium`	SS3	16	2.5e-4	0.0	73.28 ACC
		16	2.5e-4	0.05	73.43 ACC
		16	5.0e-4	0.05	73.50 ACC
		32	2.5e-4	0.05	73.30 ACC
		16	5.0e-4	0.1	**73.57** ACC
		32	2.5e-4	0.1	73.33 ACC
		32	5.0e-4	0.05	73.36 ACC
		8	5.0e-4	0.05	73.52 ACC
	SS8	8	5.0e-4	0.05	53.66 ACC
		16	5.0e-4	0.05	**60.98** ACC
		32	2.5e-4	0.05	60.52 ACC
		32	5.0e-4	0.05	60.62 ACC
		16	5.0e-4	0.1	60.70 ACC
		32	5.0e-4	0.1	60.83 ACC
	BUR	8	2.5e-4	0.0	78.75 ACC
		16	2.5e-4	0.0	78.77 ACC
		8	5.0e-4	0.05	**78.92** ACC
		16	2.5e-4	0.05	78.88 ACC
		16	2.5e-4	0.1	78.92 ACC
		32	2.5e-4	0.05	78.78 ACC
		16	5.0e-4	0.1	78.81 ACC
		32	2.5e-4	0.1	78.88 ACC
	ASA	16	2.5e-4	0.0	66.09 PCC
		16	2.5e-4	0.05	**66.37** PCC
		16	5.0e-4	0.05	65.98 PCC
		16	2.5e-4	0.1	66.23 PCC
		32	2.5e-4	0.1	66.31 PCC
	PPI	8	1.25e-4	0.0	61.82 AUC ROC
		8	2.5e-4	0.0	57.91 AUC ROC
		8	1.25e-4	0.05	**62.11** AUC ROC
		32	2.5e-4	0.05	58.68 AUC ROC
		8	1.25e-4	0.1	60.72 AUC ROC
	EPI	8	6.25e-5	0.05	68.31 AUC ROC
		16	2.5e-4	0.05	**69.90** AUC ROC
		16	5.0e-4	0.05	68.47 AUC ROC
		8	1.25e-4	0.1	68.68 AUC ROC
	HPR	16	2.5e-4	0.0	18.14 PCC
		32	6.25e-5	0.0	**19.61** PCC
		8	2.5e-4	0.1	18.81 PCC
		32	6.25e-5	0.1	19.18 PCC
`base`	SS3	8	2.5e-4	0.0	**74.84** ACC
		8	5.0e-4	0.0	73.48 ACC
		8	2.5e-4	0.05	74.77 ACC
		8	5.0e-4	0.05	66.25 ACC
		8	5.0e-4	0.1	74.83 ACC
	SS8	8	2.5e-4	0.0	**61.89** ACC
		8	5.0e-4	0.0	53.52 ACC
		8	2.5e-4	0.05	61.70 ACC
		4	2.5e-4	0.0	60.70 ACC
	BUR	4	2.5e-4	0.0	79.22 ACC
		8	2.5e-4	0.0	79.49 ACC
		8	2.5e-4	0.05	79.38 ACC
		8	2.5e-4	0.1	**79.55** ACC
	ASA	8	1.25e-4	0.0	**66.07** PCC
		8	6.25e-5	0.05	64.98 PCC
		8	1.25e-4	0.05	65.73 PCC
		4	1.25e-4	0.1	65.10 PCC
		8	1.25e-4	0.1	65.89 PCC
		8	1.25e-4	0.2	65.10 PCC
	PPI	8	6.25e-5	0.0	**61.62** AUC ROC
		8	1.25e-4	0.0	60.22 AUC ROC
		4	6.25e-5	0.05	60.78 AUC ROC
		4	1.25e-4	0.1	60.49 AUC ROC
		8	6.25e-5	0.1	59.29 AUC ROC
		4	1.25e-4	0.2	60.67 AUC ROC
	EPI	8	6.25e-5	0.0	69.47 AUC ROC
		4	6.25e-5	0.05	69.27 AUC ROC
		8	6.25e-5	0.1	69.81 AUC ROC
		8	1.25e-4	0.1	**69.51** AUC ROC
	HPR	8	6.25e-5	0.0	18.67 PCC
		8	6.25e-5	0.1	**18.72** PCC
		8	1.25e-4	0.1	18.55 PCC
		4	6.25e-5	0.2	18.58 PCC

The hyperparameters are tuned on each structural prediction task individualy on the validation set. First, all hyperparemeter combinations were tested once in a grid search. For each tasks, the four till eight best combinations were further investigated by training and validate the structural prediction tasks another three times. The mean performance for the selected hyperparameter combinations per tasks are shown here. The highest performance is shown in bold.

Table 2. *The tuned hyperparameters for the fine-tuning tasks of* `medium` *and* `base` *models.*

Model	Pre-training task	Batch size	Learning rate	Dropout rate
`medium`	SS3	16	5e-4	0.1
	SS8	16	5e-4	0.05
	BUR	8	5e-4	0.05
	ASA	16	2.5e-4	0.05
	PPI	8	1.25e-4	0.05
	EPI	16	2.5e-4	0.05
	HPR	32	6.25e-5	0.05
`base`	SS3	8	2.5e-4	0.0
	SS8	8	2.5e-4	0.0
	BUR	8	2.5e-4	0.1
	ASA	8	1.25e-4	0.0
	PPI	8	6.25e-5	0.0
	EPI	8	1.25e-4	0.1
	HPR	8	6.25e-5	0.1

The hyperparameters are tuned for each downstream structural prediction task individualy on both the converged `medium` pre-trained model and the converged `base` pre-trained model using the validation sets.

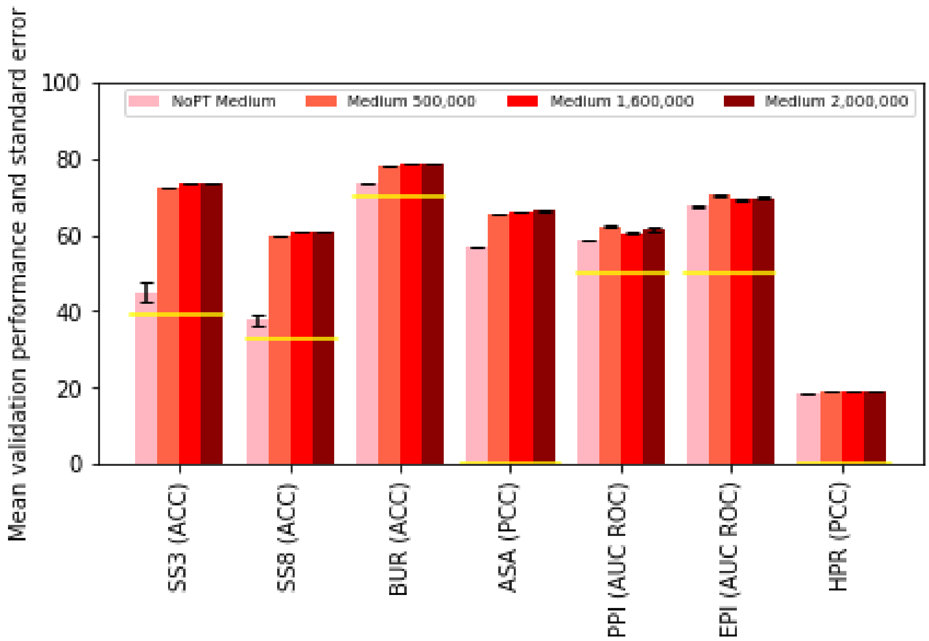

a

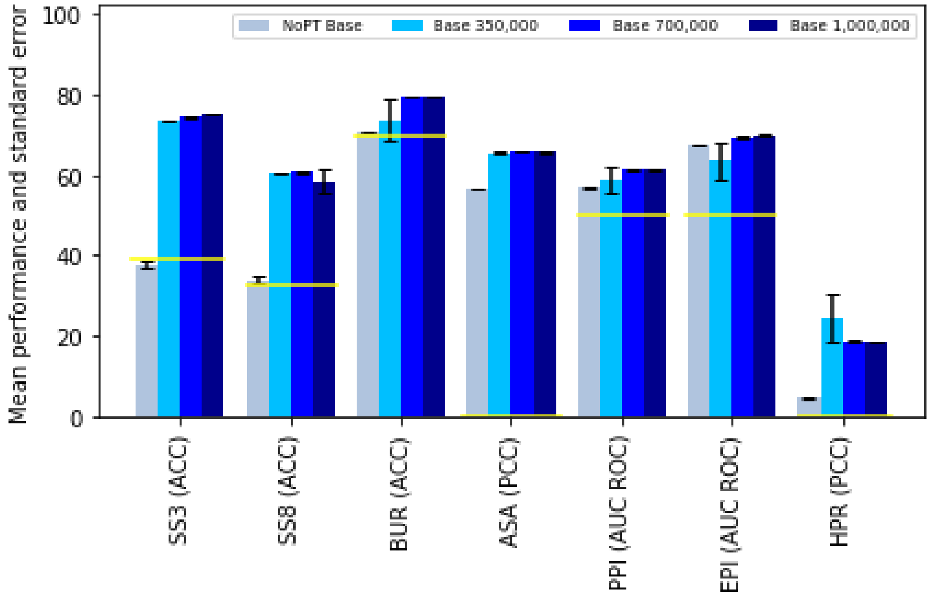

b

Figure 3. *Performance of fine-tuning tasks monitored during pre-training.* Prediction performances of the validation set, (**a**) for a medium sized model, and (**b**) for a base sized model, trained without the pre-training step (pink/grey), and on a pre-trained base size model for 350 000 (light red/blue) steps, 700 000 steps (red/blue) and 1 000 000 steps (dark red/blue).
